# The golden ratio as an ecological affordance leading to aesthetic attractiveness

**DOI:** 10.1002/pchj.505

**Published:** 2021-12-23

**Authors:** Daniela De Bartolo, Maria De Luca, Gabriella Antonucci, Stefan Schuster, Giovanni Morone, Stefano Paolucci, Marco Iosa

**Affiliations:** ^1^ Santa Lucia Foundation Rome Italy; ^2^ Department of Psychology Sapienza University of Rome Rome Italy; ^3^ Department of Bioinformatics Friedrich Schiller University Jena Jena Germany

**Keywords:** aesthetics, beauty, eye tracking, golden ratio, neuroaesthetics, psychophysics

## Abstract

The golden ratio (GR) is an irrational number (close to 1.618) that repeatedly occurs in nature as well as in masterpieces of art. The GR has been considered a proportion perfectly representing beauty since ancient times, and it was investigated in several scientific fields, but with conflicting results. This study aims at investigating if this proportion is associated with a judgment of beauty independently of the type of the stimulus, and the factors that may affect this aesthetic preference. In Experiment 1, an online psychophysical questionnaire was administered to 256 volunteers asked to choose among three possible proportions between the parts of the same stimulus (GR, 1.5, and 1.8). In Experiment 2, we recorded eye movements in 15 participants who had to express an aesthetic judgment on the same stimuli of Experiment 1. The results revealed a slight overall preference for GR (53%, *p* < .001), with higher preferences for stimuli representing humans, anthropomorphic sculptures, and paintings, regardless of the cultural level. In Experiment 2, a shorter dwell time was significantly associated with a better aesthetic judgment (*p* = .005), suggesting the possibility that GR could be associated with easier visual processing, and it could be hence considered as a visual affordance.

## INTRODUCTION

The sense of beauty represents a very addressed and discussed topic in the field of psychology (Ramachandran & Hirstein, 1999), and in the last two decades, neuroaesthetics have become an important field of research for neurophysiologists and neuropsychologists (Bundgaard et al., 2017; Zeki, 1999a, 1999b) interested in the cognitive and perceptual processes involved in the perception of beauty (Bundgaard, 2015). Recent studies exploited the eye tracking methodology to objectively analyze the “salient” properties of an object that may capture the beholder's attention (Bundgaard, 2015; Chassy et al., 2015; Chenier & Winkielman, 2018; Kumar & Garg, 2010; Reber, 2012), using electroencephalography (Kong et al., 2015; Zhang et al., 2020; Zhang & Deng, 2012) and functional magnetic resonance imaging (Di Dio et al., 2007; Di Dio et al., 2011) to analyze the brain areas and relevant processes involved in the perception of attractive and beautiful stimuli. Among the properties associated with attractiveness, researchers found symmetry, harmonic proportions, balance, contrast, and a proper easiness/complexity relationship (Fink & Penton‐Voak, 2002).

**FIGURE 1:**
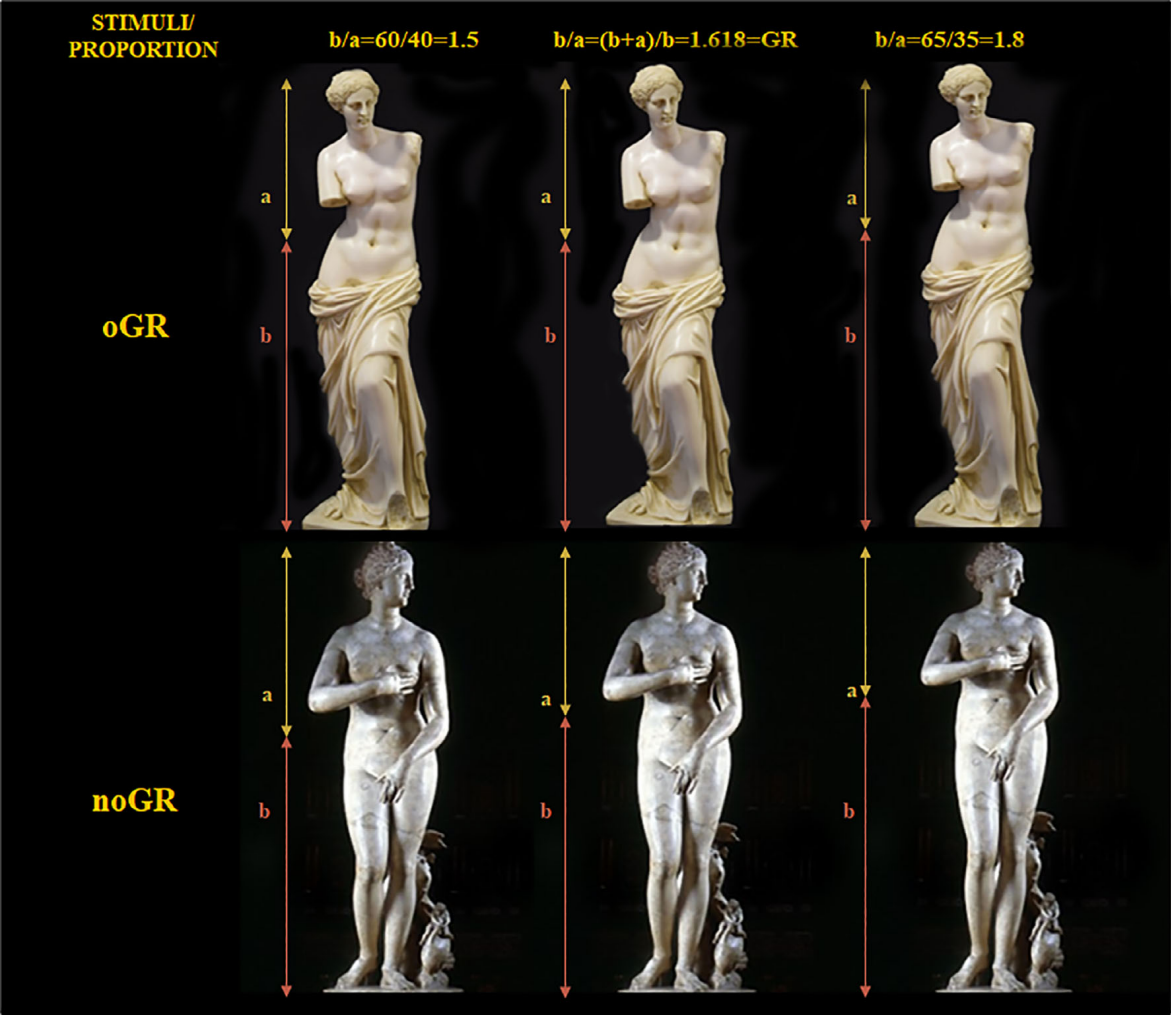
The stimuli modification procedure reported for two exemplificative stimuli: The Venus of Milo (above, originally in golden rule [GR], oGR) and the Venus de' Medici (below, not originally in GR, noGR). The GR refers to the ratio between b (from the ground to the navel) and a (from the navel to the top of the head). In the case of Venus of Milo, the original version was compared to two experimental altered versions in which b/a was 1.5 (on the left) or 1.8 (on the right). The Venus of Medici has originally a ratio b/a = 1.5, and the version in GR and R1.8 were digitally created

A particular proportion linked to harmony and studied in aesthetics is the golden ratio (GR; Iosa et al., 2018). From a geometric point of view, it is the mathematical solution of dividing a segment in a manner that the ratio between the shorter part to the longer one of the segments is equal to the ratio between the longer part and the whole segment. This problem has only a solution in which the above ratio is the irrational number φ = 1.6180… (Figure 1).

Since the 5th century BCE, this number occurs in the artworks of many artists who have considered the GR as a beautiful and pleasant proportion (Gamwell, 2015). Polykleitos sculptured the Doryphoro with the ratio between the total height and the distance of the navel from the ground equal to the GR; the same ratio was respected by Alexandros of Antioch when he sculptured the Venus de Milo. Phidias supervised the creation of the façade of the Parthenon as inscribed in a golden rectangle, namely with the ratio of basis and height of the rectangle equal to the GR (Iosa et al., 2018).

Since the 19th century, psychologists have been testing if the GR led to a subjective experience of beauty supported by objective evidence. However, conflicting results were found among studies: GR seemed to be the preferred proportion if the stimulus was a rectangle (Fechner, 1865), a triangle (Witmer, 1893), partially if it was an ellipse (Fechner, 1865), a sculpture (Di Dio et al., 2007; Di Dio et al., 2011), or related to human faces (Danikas & Panagopoulos, 2004; Kwart et al., 2012). Other researchers questioned the different methodologies with which these results were obtained (Godkewitsch, 1974; Piehl, 1976). In particular, it was questioned whether GR often emerged as the average response and median ranking but not as the modal, most selected, preferred ratio (Angier, 1903; Nienstedt Jr & Ross, 1951).

Till now, only reviews compared the potential preferences for GR among different types of stimuli, but combining data obtained in different studies, carried out with different methodologies, and using different parameters to assess aesthetic preferences (Bejan, 2009; Green, 1995; Iosa et al., 2018). Russell (2000) compared in the same experiment geometric stimuli versus paintings, finding GR was preferred in the former and not in the latter, in contrast with the fact that GR has been found as a feature of many artistic paintings (de Campos et al., 2015; Iosa et al., 2018). Some studies also suggested a possible confounding role played by cultural level of the interviewed subjects (Berlyne, 1970; Thompson, 1946).

The literature lacks studies investigating the preference for the GR comparing different classes of stimuli and analyzing if this preference may depend on education, artistic, or mathematical experiences.

The aims of this study were hence to investigate if the golden proportion is or not preferred, and if this eventual preference could depend on the type of the stimulus or on the cultural level of the observers (Experiment 1). Then, we aimed at investigating if some parameters of gaze movements, quantitatively assessed by eye tracking, could be associated with the aesthetic judgments when the stimulus has a golden proportion (Experiment 2).

This study was conducted according to the Declaration of Helsinki for research on human beings. It was approved by an independent local ethics committee of the Santa Lucia Foundation (CE /PROG660). For the first experiment the participants were recruited online, and their data were treated anonymously according to the Legislative Decree of 30 June 2003, n. 196, art. 13. The participants read all the information relating to the questionnaire administered online and gave their consent before proceeding with the compilation. For the second experiment, the participants were enrolled in the Laboratory of Developmental Dyslexia Research Laboratory located at the Santa Lucia Foundation in Rome. The participants gave their written consent before proceeding with the experiment.

## MATERIALS AND METHODS OF EXPERIMENT 1

### Participants

A sample of 256 participants accepted to complete an online administered questionnaire on a personal computer. All of them were normal or corrected‐to‐normal vision. The average age of subjects was 34.2 ± 10.8 years, their average schooling was of 16.8 ± 2.9 years, and 167 participants were female.

### Procedure

A computerized online questionnaire was administered to participants, according to psychosocial and clinical research methodology (Regmi et al., 2016). Data were treated anonymously. The questionnaire was based on the method of “absolute judgment in psychophysics”: it consists in administering stimuli divided in couples, asking the subject to select the most liked. This method of choice is commonly used to identify, if it exists, a relative order of pleasantness (Wever & Zener, 1928). Participants were asked to complete the questionnaire in a comfortable position in front of a PC monitor rather than a smartphone to avoid small displays. The online questionnaire consisted of two parts. In the first one, participants were asked to report demographic data (age, sex), their education (highest degree), and to self‐evaluate their cultural skills (because GR could be studied in the fields of mathematics, visual arts, and music, the self‐assessed knowledge of these disciplines were separately assessed). The second part contained the experimental paradigm formed by 17 stimuli, each one replicated in three different proportions, forming 51 items of coupled stimuli. In each couple, two images were simultaneously presented on the screen and subjects were asked to choose the image they liked the most by clicking with the mouse on the preferred picture. The left–right position of the three proportions on the screen were randomly balanced among the trials.

### Stimuli

The GR has been traced in the human body proportions, artistic sculptures, and paintings with which a sense of beauty is usually associated; the visual stimuli included in this study therefore included colored images of human figures (Di Dio et al., 2007), images of anthropomorphic sculptures (Di Dio et al., 2011), and images of paintings (Iosa, 2018). In addition, stimuli on which a preference for the GR has been reported in previous studies have been included, such as geometric figures (Berlyne, 1970) and bisected lines (Benjafield & Adams‐Webber, 1976). Then, the image of Leonardo's Vitruvian Man drawing was included given its notorious golden proportions. Finally, virtual humanoid avatars were also inserted to provide more recent replications of humanoid figures based on virtual tools (designed using an open‐source program Make HumanTM for 3D humanoid prototypes) (Bastioni et al., 2008). The stimuli were chosen by balancing the numbers of those already known to be originally in GR (oGR, such as Venus de Milo; Iosa et al., 2018) and stimuli of the same categories not originally in GR (noGR, such as Venus de' Medici, Figure 1), while the geometric and virtual stimuli, being created ad hoc, had no original proportion. Each figure was divided according to a specific ideal line that was the horizontal line passing for the navel of the anthropomorphic figures (photographs, paintings, sculptures, or virtual reproductions of humans) or the vertical line dividing the scene in two parts for horizontal paintings. The proportions between the vertical dimensions of the obtained two parts for human figures and those between the two horizontal dimensions for horizontal paintings were altered to obtain in total three stimuli in proportions of the GR, 1.5, or 1.8. The same proportions were recreated for geometric stimuli selecting the cut point in line section or altering the proportion of basis and height of the rectangle. This procedure is shown graphically in Figure 1.

As in previous studies (Di Dio et al., 2007; Di Dio et al., 2011; Fechner, 1865), each stimulus was presented in three possible versions having the following main proportion between two main parts of the picture (with a tolerance of 0.5%): 61.8:38.2% (ratio = 1.618, approximately equal to GR), 65%–35% (ratio = 1.8, furtherly called R1.8) or 60:40% (ratio = 1.5, R1.5). The other two proportions were chosen in accordance with previous studies (Haines & Davies, 1904) and with the fact that subjects are often exposed also to them (Benjafield & Adams‐Webber, 1976). In fact, R1.8 is close to the ratio of television and computer monitors (16:9 = 1.78, or 1920:1080 pixels = 1.78), and it was also one of the ratios used in previous studies (Di Dio et al., 2007; Di Dio et al., 2011); whereas R1.5 was chosen because some studies suggested that the actual preferred proportion is 3:2 (=1.5) and not GR (Davis, 1933), as also occurred for the ellipses of the study of Fechner (1865).

The 17 stimuli are reported in Table 1. Anthropomorphic figures (humans, humanoids, sculptures, and Vitruvian Man) were shown as entire standing figures, and the main proportion considered was the ratio between the total height and the distance of the navel from the ground, according to previous studies (as shown in Figure 1; Davis & Altevogt, 1979; Di Dio et al., 2007). For paintings, the main proportion was that related to GR (de Campos et al., 2015) or another clear horizontal division between two main parts of the painting. The three couples of comparisons (GR vs. R1.8, GR vs. R1.5, R1.8 vs. R1.5) multiplied for the 17 stimuli resulting in the 51 items of the questionnaire.

**TABLE 1:** List of stimuli

Stimulus	Category	Main proportion	Original proportion
1. Woman	Photographs of real humans	Ratio between the distance of the navel to the ground and that between the navel and the top of the head	62–38% (oGR)
2. Man			62–38% (oGR)
3. Woman			58–42% (noGR)
4. Man			65–35% (oGR)
5. Venus de Milo	Photographs of sculptures		62–38% (oGR)
6. Doryphoros			62–38% (oGR)
7. Venus de' Medici			60–40% (noGR)
8. David			59–41% (noGR)
9. Virtual woman	Virtual avatars		‐
10. Virtual man			‐
11. Vitruvian Man	Drawing		62–38% (oGR)
12. The Flagellation of Christ	Paintings	Ratio between right and left parts with respect to a main element of the scene	62–38% (oGR)
13. The Creation of Adam			62–38% (oGR)
14. The Calling of St. Matthew			60–40% (noGR)
15. Undergrowth with two figures			64–36% (noGR)
16. Section of a horizontal line	Geometric figures	Ratio between two parts	‐
17. Rectangle		Basis/height	‐

*Note*: For the artistic stimuli the authors were: (5) Alexandros, (6) Polykleitos, (7) Cleomenes, (8 and 13) Michelangelo Buonarroti, (11) Leonardo da Vinci, (12) Piero della Francesca, (14) Caravaggio, and (15) van Gogh.

### Statistical analysis

All analyses were performed using IBM SPSS Statistics Version 23.0. The sample size for Experiment 1 was determined by computing estimated statistical power, based on the results of a previous study (Di Dio et al., 2007). Fixing the alpha level at .05 and power of the test at .80, we were required to enroll at least 255 subjects.

The percentage preference for GR, R1.5, and R1.8 proportions were computed for each stimulus and averaged for each stimulus category. Comparisons were performed using the chi‐squared test. Because each proportion was compared to another two, the possibility of finding a statistically significant results was divided by 2 applying Bonferroni correction. So, the alpha level of statistical significance was set at .025. Odds ratio (OR) and 95% confidence interval (95% CI) were also computed. The correlations of the aesthetic preference with demographic variables and cultural skills were tested using Spearman's rank correlation coefficient.

## RESULTS OF EXPERIMENT 1

Table 2 shows the percentages of participants' choices for the three presented comparisons. As shown in the last columns of Table 2, GR preferences overcame the 50% in five out of six categories and in 14 out of 17 stimuli. Among these, the significant preferences found in favor of GR were related to photographs of humans (54%, *p* = .005), sculptures (53%, *p* = .029), paintings (55%, *p* = .004), and Vitruvian Man (58%, *p* = .015). Preference was only close to significance for virtual humanoids (54%, *p* = .057). For geometric shapes, GR was chosen in 48% of trials, a percentage not significantly different from those of other proportions (*p* = .353). The sum of all responses highlighted a mean significant preference for GR (53%, *p* < .001).

**TABLE 2:** Percentages of choices and *p*‐values related to chi‐square among the three comparisons between the ratios (*p*‐values are in bold are lower than alpha‐level reduced for Bonferroni's correction; special character Φ was added to significant results in favor of GR ratio)

Stimulus	GR vs. R1.8		GR vs. R1.5		R1.5 vs. R1.8		Mean % for GR	
	%	*p*	%	*p*	%	*p*	Stimulus	Category
1. oGR woman	62.5%	**<.001** ^**Φ**^	64.5%	**<.001** ^**Φ**^	38.3%	**<.001**	63.5%	54.4%
2. oGR man	62.9%	**<.001** ^**Φ**^	52.0%	.532	53.5%	.261	57.5%	
3. noGR woman	40.6%	**.003**	39.1%	**<.001**	52.0%	.532	39.9%	
4. no GR man	60.5%	**.001** ^**Φ**^	53.1%	.317	55.1%	.104	56.8%	
5. oGR Venus	64.5%	**<.001** ^**Φ**^	49.6%	.901	57.0%	**.024**	57.1%	53.4%
6. oGR Doryphoros	52.3%	.453	53.9%	.211	58.6%	**.006**	53.1%	
7. noGR Venus	47.8%	.532	55.5%	.08	66.4%	**<.001**	51.7%	
8. noGR David	67.2%	**<.001** ^**Φ**^	36.7%	**<.001**	60.2%	**.001**	52.0%	
9. Neutral virtual woman	63.3%	**<.001** ^**Φ**^	45.3%	.134	65.0%	**<.001**	54.3%	54.2%
10. Neutral virtual man	76.6%	**<.001** ^**Φ**^	31.6%	**<.001**	71.5%	**<.001**	54.1%	
11. oGR Vitruvian Man	73.0%	**<.001** ^**Φ**^	42.2%	**.012**	71.5%	**<.001**	57.6%	57.6%
12. oGR Della Francesca	59.8%	**.002** ^**Φ**^	57.0%	**.024** ^**Φ**^	58.2%	**.009**	58.4%	54.6%
13. oGR Michelangelo	49.2%	.803	52.7%	.382	41.4%	**.006**	51.0%	
14. oGR Caravaggio	60.2%	**.001** ^**Φ**^	58.6%	**.006** ^**Φ**^	52.7%	.382	59.4%	
15. noGR van Gogh	43.0%	**.024**	55.9%	.061	45.7%	.169	49.5%	
16. Neutral line section	47.3%	.382	42.6%	**.018**	48.0%	.532	45.0%	48.0%
17. Neutral rectangle	47.3%	.382	54.7%	.134	40.2%	**.002**	51.0%	

*Note*: In the last two columns the values of the mean golden rule (GR) were reported for each stimulus and each category of stimuli.

Many statistically significant differences were found regarding the comparison between GR and R1.8 proportion (12 out of 17 stimuli). Indeed, 10 comparisons were in favor of GR (57.5% vs. 42.5%, *p* = .018), while only two were in favor of the R1.8 proportion (the painting of a woman and the painting of van Gogh, both noGR).

When compared with the R1.5 proportion, the preference for GR was reduced: it was significantly in favor of GR for three stimuli (the picture of a woman, Della Francesca's painting, both oGR stimuli, and for Caravaggio's painting, an noGR stimulus), and in favor of R1.5 proportion for five stimuli. The overall preference was in line with chance: 50% for GR and 50% for R1.5 proportion. The comparison R1.5 versus R1.8 showed a not significant preference for R1.5 (*p* = .134).

**FIGURE 2:**
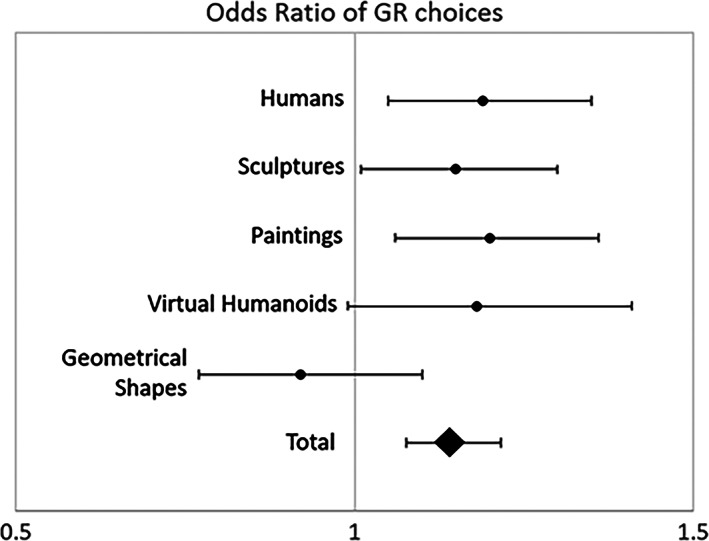
Forest plot of odds ratios (OR) and relevant 95% confidence intervals estimated for stimulus originally in golden rule (GR) with respect to chance (OR = 1 corresponds to no association between object's proportion and subject's choice) for the categories of the stimuli

Figure 2 shows the comparisons of preferences for GR with respect to the other two proportions, finding statistically significant differences for photographs of humans (OR = 1.19, 95% CI = 1.05–1.35, *p* = .005), sculptures (OR = 1.15, 95% CI = 1.01–1.30, *p* = .029), and paintings (OR = 1.20, 95% CI= 1.06–1.36, *p* = .004), but for neither virtual humanoids (OR = 1.18, 95% CI = 0.99–1.41, *p* = .057) nor geometric shapes (OR = 0.92, 95% CI = 0.77–1.10, *p* = .353).

The total preference for GR was found related neither with age nor with any cultural skills (age: *p* = .574, schooling time: *p* = .860, self‐assessed art knowledge: *p* = .173, math knowledge: *p* = .059, music knowledge: *p* = .209). Dividing the results for the categories of stimuli, the preference for GR was found significantly correlated with self‐assessed artistic (*p* = .035) and mathematical (*p* = .023) knowledge for human photographs, whereas GR preference for sculptures was found significantly correlated with artistic (*p* = .015) and musical (*p* = .020) knowledge.

## MATERIALS AND METHODS OF EXPERIMENT 2

### Participants

Fifteen healthy subjects (nine female) with an average age of 28.7 ± 6 years and average schooling of 18.9 ± 2.2 years, with normal or corrected‐to‐normal vision, and naïve to the purpose of the experiments were enrolled. The sample size was chosen in accordance with the data of a previous study (Di Dio et al., 2007).

### Stimuli and procedure

The same set of 51 stimuli of Experiment 1 was used, but presented one by one during individual eye movement recording sessions in which the subject sat in front of a monitor at a viewing distance of 57 cm. The rectangular stimuli subtended a viewing angle of approximately 8.0 × 10.0 and 4.0 × 8.0 degree**s** (width by height), according to a previous study (McManus et al., 2010).

The modification of ratio was made without altering the visual angle for each stimulus; this allowed comparison of the same stimulus in the three experimental ratios maintaining the same visual angle.

The participants were asked to watch each stimulus and to express a verbal judgment using a 10‐point Likert scale (from 1 = *strongly dislike* to 10 = *strongly like*). Before the experiment started, each subject was instructed about the task, placed in front of the screen using a head support, and trained to carry out the experiment through a pilot including a nine‐point calibration procedure and a set of 10 stimuli that were different but homologous to the stimuli of the main experiment.

### Setting and apparatus

The experiment was carried out on a desktop computer. Stimuli had a 300 dpi resolution and were presented at 85 Hz on a 17‐inch CRT monitor, with a screen resolution of 1024 × 768 pixels. Eye movements were recorded in binocular vision from the dominant eye via an EyeLink 1000 eye‐tracker (SR Research), sampling at 1000 Hz. Appearance of the stimulus on the screen was triggered by fixation of a cross outside the stimulus area. Subjects' vocal responses scoring the aesthetic judgments were digitally recorded by a Shure microphone, a pre‐amplifier, an E‐MU sound card, and an ASIO driver, which was interfaced to the eye tracker by Eye Link software.

### Data processing

**FIGURE 3:**
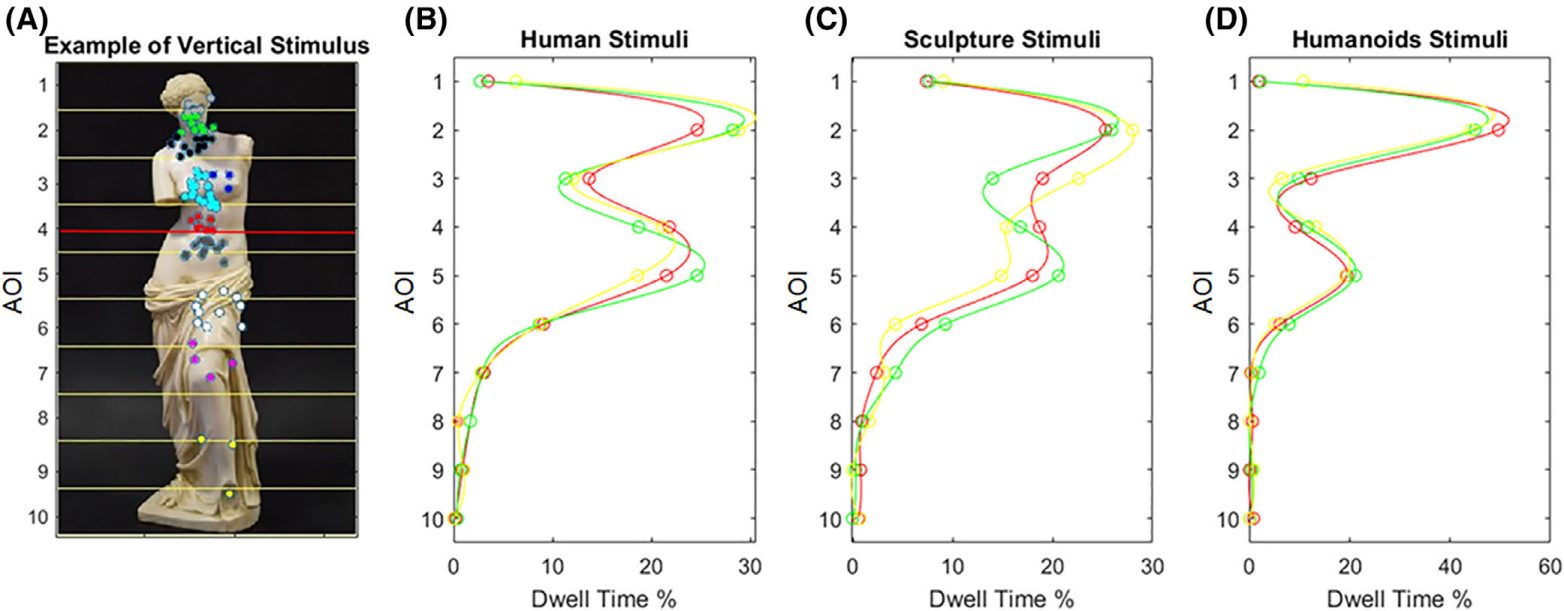
Graphical representation of how fixations were distributed among the vertical anthropomorphic stimuli. In the first panel (A) the Venus of Milo, as example, in its original golden rule (GR) proportion with a red line marking where the alteration of ratio was done. Colored points represent groups of fixations spatially closed. In the other panels we reported the percentage of dwell time spent in each area of interest (AOI) normalized on the total dwell time for all the stimuli belonging to human figures (B), anthropomorphic sculptures (C) and virtual humanoids (D) category, comparing the three experimental proportions: GR (red), R1.5 (green), and R1.8 (yellow)

**FIGURE 4:**
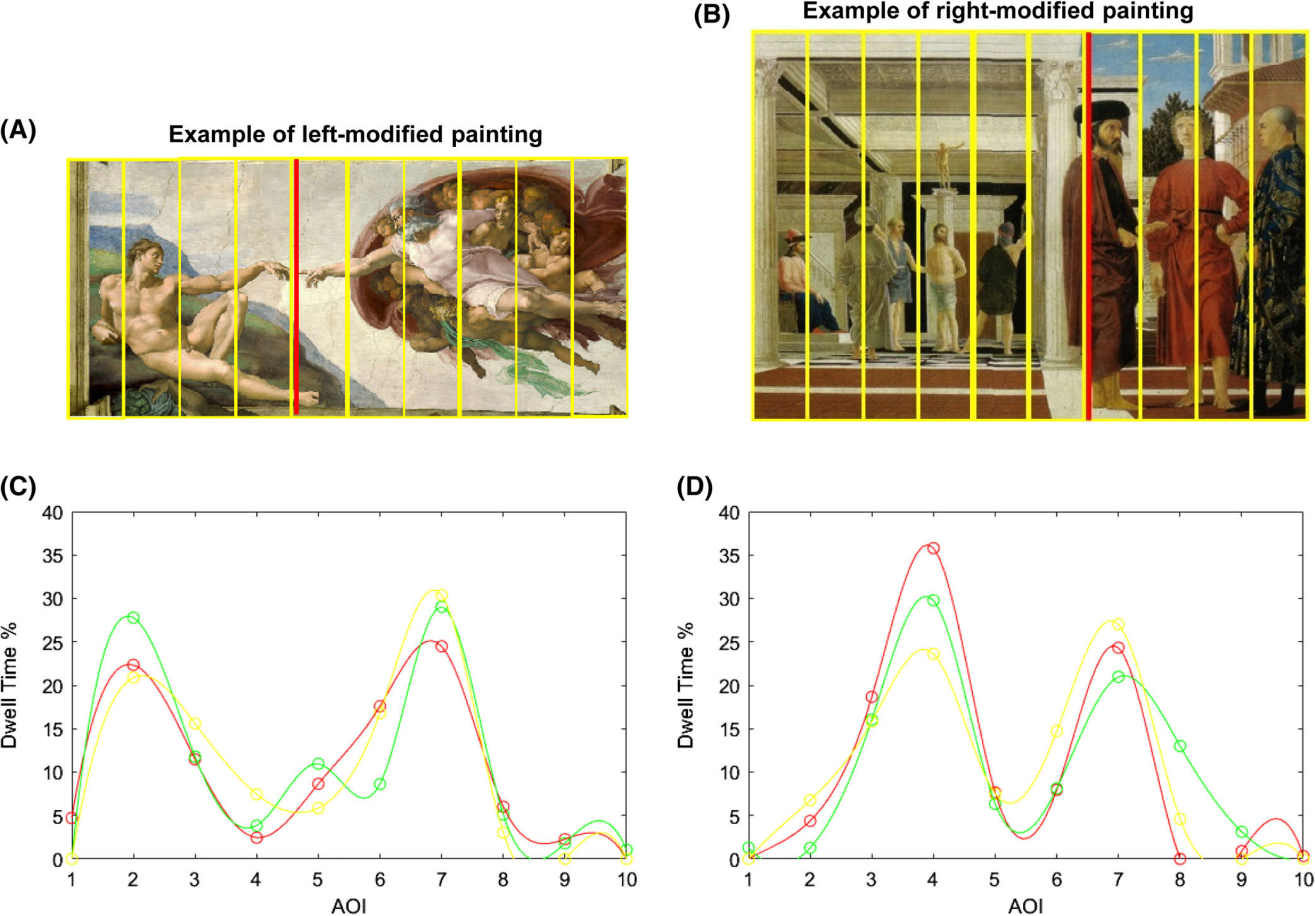
Example of horizontal stimuli in which ratio alteration (performed in correspondence of red vertical lines) occurred in the left (panel A) or right (panel B) side. Panels (C) and (D) show the distribution of dwell times for the paintings on the left in each interest area (defined by the yellow vertical lines in panels A and B), comparing the three experimental proportions: GR (red), R1.5 (green), and R1.8 (yellow)

Eye movement data were processed via EyeLink Data Viewer software (SR Research). Eye blinks were automatically signaled by the software and manually discarded during the offline visual inspection carried out by the experimenter over each single trial. Fixation positions were measured and referred to areas of interest (AOI) for each stimulus trial according to 10 horizontal (for portrait stimuli, as in Figure 3) or vertical (for landscape stimuli, as in Figure 4) AOI of equal size. In particular, for each trial, the specific AOI in which the modification of ratio occurred was designated as AOIm. The dwell time per AOI (AOI dwell time) and the dwell time per trial (i.e., the total stimulus dwell time, TOT dwell time) were computed, along with the percentage ratio of time spent fixating the AOI over the time spent fixating the whole stimulus (% AOI/TOT dwell time).

### Statistical analysis

The statistical analyses were performed using IBM SPSS v.23 statistical software. Data results were not normally distributed (Shapiro–Wilk test), and hence non‐parametric statistics were used. Friedman's analysis was used to detect differences among the three conditions (GR, R1.5, and R1.8), and Wilcoxon test for paired sample was used for post hoc analysis between conditions. Spearman's rank correlation coefficient was computed to verify possible comparisons among these parameters and the given aesthetic judgment. The alpha level of significance was set at .05, but Bonferroni's correction was applied for post hoc multiple comparisons, reducing the alpha level at .025 for post hoc tests and at .017 for correlations.

## RESULTS OF EXPERIMENT 2

The aesthetic judgments given in Experiment 2 were analogous to those found for Experiment 1. The judgments assigned to the GR stimulus resulted slightly but significantly higher in GR (6.71 ± 1.36) than in R1.5 (6.45 ± 1.45, *p* = .046), whereas those of R1.8 showed higher variability (6.34 ± 1.61, *p* = .056). In general, the score assigned to GR was higher than the mean score assigned to the other two proportions (*p* = .003), in particular for human figures (6.87 ± 1.62 vs. 6.54 ± 1.55, *p* = .015). Figure 3 shows an example of fixation distribution on a vertical stimulus in terms of average percentages of AOI dwell time for GR, R1.5, and R1.8.

As shown in Figure 3, the face (AOI2) was the most observed, with another peak around the navel. This double‐peak pattern was evident for human photographs, sculptures, and virtual humanoids. For the photographs of humans and sculptures, when the stimulus was altered, the gaze moved accordingly: if the height of the navel was lowered, as in R1.5, the eyes moved downward and time spent in the lower AOI4 increased; when the height of the navel was lifted up, as in R1.8, the eyes moved upward, and time spent in AOI3 increased. This pattern was not observed for virtual humanoids, for which the face was observed for more than half of the total dwell time.

Friedman's test highlighted significant differences among GR, R1.5, and R1.8 regarding dwell time spent to explore the AOIm (chi‐squared = 6.845, *p* = .033) and regarding the percentage AOIm dwell time/TOT dwell time (chi‐squared = 6.345, *p* = .042), but not in the TOT dwell time (chi‐squared = 0.808, *p* = .668). Post hoc analyses with Wilcoxon test showed a statistical difference for AOIm dwell time only regarding the comparison between R1.8 and R1.5 (*p* = .009). Furthermore, the percentage of AOIm compared to the total was different between GR and R1.5 (*p* = .009) as well as between R1.5 and R1.8 (*p* = .008).

**TABLE 3:** Spearman's rank correlation coefficient (*R*) and relevant *p*‐values (in bold if statistically significant) computed between aesthetic judgment and temporal parameters of eye movements (Experiment 2)

Eye movement parameters		R1.5			GR			R1.8		
		AOIm	TOT	AOIm/TOT (%)	AOIm	TOT	AOIm/TOT (%)	AOIm	TOT	AOIm/TOT (%)
Judgment Correlation	R	−.108	−.018	−.076	−.175	−.112	−.175	−.059	−.069	−.049
	*p*	.085	.774	.225	**.005**	.074	**<.001**	.350	.275	.436

*Note*: AOIm dwell time stands for the average value of fixation time spent in exploring the area of interest in which the stimulus ratio has been modified; TOT dwell time refers to the total fixation time spent exploring the stimulus; AOIm/TOT (%) is the ratio between the average fixation time spent exploring the AOIm with respect to the total dwell time expressed in percentage.

Table 3 reports the correlations among the eye movement parameters and the reported esthetic judgments, resulting in significant correlation to AOIm time and AOIm/TOT dwell time percentages only for GR stimuli.

Table 3 reports the correlations among the eye movement parameters and the reported aesthetic judgments. The aesthetic judgment significantly correlated to AOIm time and AOIm/TOT dwell time percentage only for GR stimuli.

Finally, the %AOI/TOT dwell time grouped according to the category of the stimulus showed statistically significant differences (*p* < .005) only for painting stimuli among the three proportions.

Figure 4 shows these results, with a trend similar to those of Figure 3, but along the horizontal axis. Again, the pattern followed the modification of the ratio between the two parts of the figure. In the left‐modified stimuli (as the Creation of Adam), significant differences were found for AOI3 (chi‐squared = 13.273, *p* = .001) with higher percentage dwell time for R1.8, as well as AOI5 for R1.5 (chi‐squared = 12.991, *p* = .002). In The Flagellation of Christ, the AOIm was on the right: the AOI7 received more fixation time when the division was put in its middle as in R1.8, than in R1.5, for which the division moved in AOI6.

## DISCUSSION

The main purpose of this study was to establish whether the presence of the GR may favor the perception of beauty in aesthetic judgments and which factors might influence it, clarifying the main controversies found in previous studies (Benjafield & Adams‐Webber, 1976; Berlyne, 1970; Boselie, 1984; Davis & Jahnke, 1991; Di Dio et al., 2007; Fechner, 1865; Green, 1995; Witmer, 1893; Zeising, 1855).

First of all, we found a slight but significant preference for GR, but its entity depended on the category of the stimulus. This preference was statistically significant for human photographs, in both the experiments, and for sculptures and paintings in Experiment 1 (being only close to a significant threshold for virtual humanoid figures).

Conversely, we did not find a significant preference for GR in geometric stimuli. These findings are in line with some previous results (Di Dio et al., 2007; Di Dio et al., 2011) but in contrast with other studies finding a preference for GR also for geometric figures (Fechner, 1865; Russell, 2000). There are many possible explanations for the high variability among studies related to the possible preference for golden rectangles. First of all, it could be related to different methodologies related to how central tendency was computed: by means of mean, median, or modal values, with the GR that could emerge as mean value even if it was not the most frequent choice, as also previously suggested (Green, 1995). Indeed, in contrast to previous research on lines and geometric shapes' aesthetic preference, recent studies used more ecological experimental paradigms including natural and anthropomorphic figures, highlighting that geometric simple stimuli could be poorly attractive for our culture (Di Dio et al., 2007; Di Dio et al., 2011). Furthermore, with respect to the study of Fechner (1865) and successive ones, subjects are now more often exposed to rectangular shapes different from GR, such as those of television screens, monitors, or smartphones having proportions closer to 1.8 (16:9 = 1.78, or 1980:1020 pixels = 1.78), or also paper sheets closer to 1.5 (A4: 1.41).

The GR is often present in many natural stimuli (Iosa et al., 2018). Indeed, subjects might prefer stimuli recognized as original, as in our study occurred for human photographs, with GR preferred for stimuli originally in GR (oGR vs. noGR), despite participants not knowing which was the original stimuli.

The golden section was conceivably identified by anatomical Greek investigations in the 5th century BCE (as in the canon of Protagoras) with the idea to reproduce the harmony of the human body in the anthropomorphic sculptures and then in other artworks (Haines & Davies, 1904; Iosa et al., 2018). It should be considered that anthropomorphic stimuli have their own symmetry given by the right–left relationship along the horizontal axis, whereas the golden section defines the vertical harmony of a standing human being. Hence, the GR preference could occur more frequently on the vertical than horizontal axis. Despite our studies not investigating this aspect, it should be noted that the rectangles of Fechner (1890) had the longer segment along the vertical axis, whereas in our and other studies (McManus, 1980; Russell, 2000) the longer dimension of rectangles and that of the bisected line was the horizontal one.

Despite this, Renaissance artists provided a horizontal division of their paintings according to the golden section, such as in The Flagellation of Christ and The Creation of Adam (de Campos et al., 2015), with these stimuli that can be mentally divided into two parts and visually explored horizontally. A slight preference for GR was found in our paintings (54.6%), quite independent if the original stimuli was or not in golden proportion. Wölfflin (1994) questioned the preference for GR when observing an abstract rectangle because it should be related to a mental comparison of the lengths of basis, height, and their sum, again claiming that GR may “present an average measure conforming to man”.

This hypothesis put in relationship the GR with “objective beauty”, because it was related to a harmonic property intrinsic to the stimuli, but there is also an alternative hypothesis, reported in the so‐called constructal theory, referring to “perceptual beauty” suggesting that the preference for GR was related to the hypothesis that visual system scans the world approximatively in golden proportion (Bejan, 2009). Di Dio et al. (2007) identified also the “subjective beauty” driven by one's own emotional experiences and related to the activation of the amygdala, whereas the objective beauty related to the intrinsic properties of the stimulus is based on a joint activation of sets of cortical neurons of lateral occipital cortex, parietal cortex, and anterior insula. All these processes could be not mutually exclusive and cohabit, forming our sense of beauty.

This integrated approach works even better considering GR as an affordance, and hence a property intrinsic to the stimulus that is recognized by our visual system and favoring the visual process. In fact, Gibson proposed the theory of affordances, suggesting that subjects noted the features of a stimulus that may constitute functional relations between the stimulus and the perceiver (Gibson, 2014; Lobo et al., 2018). The abstraction of geometric figures and even of virtual humanoids might hence have mitigated this preference, in accordance with the ecological approach suggested by Gibson for the visual system (Gibson, 2014). The cultural level of subjects did not seem to influence the general preference for GR. This result could be read in conjunction with previous findings that the aesthetic judgment of beauty was mainly independent by sociocultural factors, intelligence, personality, and age (Eysenck & Tunstall, 1968). However, in our study, culture was assessed in terms of scholarly level, but different cultures were not analyzed, and further studies could investigate the preferences for GR comparing subjects of Western versus Eastern Countries.

When we analyzed single categories of stimuli, the preference for GR in human photographs and sculptures was related to art knowledge. These two types of stimuli have in common the same criterion for GR: the ratio between the distance of the navel from the ground and that between the navel and the top of the head. Virtual humanoids had the same proportion, but the judgments about them seemed to be not affected by cultural level. Probably, this judgment mainly depended on the realism of their faces, as shown by the long time spent by observers looking at the virtual faces in Experiment 2. Furthermore, being human bodies on average in GR, humans might have reflected this proportion in the concept of beauty, and it led to the adoption of this parameter also in some classical artistic masterpieces, starting from anthropomorphic sculptures (Hernández‐Castro, 2007).

Human beings may associate, more or less consciously, the concept of beauty to their own body proportions (Burrell, 1932), as is the case with the preference of symmetric figures that could be related to the symmetry of the human body (Evans et al., 2012; Huang et al., 2018; Mühlenbeck et al., 2016). As well as symmetry being considered as an affordance in accordance with the ecological approach suggested by Gibson for vision (2014), the GR that can be considered as a symmetry of higher order (Liu & Sumpter, 2018) could also be another visual natural affordance, preferred by humans and put in artworks by artists.

In our study, the proposed paintings have the longest dimension horizontal; indeed, the golden ideal division was along the horizontal axis. A slight preference (54.6%) was found for GR, quite independently if the stimulus was or not originally in GR. With respect to the geometric figures, participants could perceive the harmony of a scene ideally divided in two parts according to the GR, even if this scene is represented with the horizontal length longer than the vertical. Considering that for symmetry there is a perceptual sensitivity to the orientation of the stimulus (1905; Fisher & Bornstein, 1982; Mach, 1883), and that there are no studies that directly investigated the relationship between GR and the orientation of the stimulus, future studies should investigate whether there is a dependence on the orientation with which it is presented the stimuli and the possible preference for GR.

Despite our study lacking this direct comparison, the bimodal distribution of dwell time found in Experiment 2 for human figures for which the golden section regards the vertical axis, are similarly replicated for the paintings in which it regards the horizontal axis. In general, the results of Experiment 2 were found in accordance with those of Experiment 1 and seem to confirm the hypothesis of GR as an affordance. In Experiment 2, we found no significant differences between the three proportions related to the total exploration time, but also some significant differences with respect to specific areas of interest. Further, analyzing the dwell times, significant differences among GR, R1.5, and R1.8 were found regarding the exploration of the AOIm, that is, the specific AOI where the image modification was performed. A significant negative correlation was found between the AOIm dwell time and the aesthetic judgment only for GR stimuli. This could indicate that the subject does not need to observe that area for too long to judge it as beautiful if it is in golden proportion.

Despite previous studies showing that the aesthetic appreciation of beauty is not based on an “immediate” response and may even require more time to discriminate the main characteristics of the stimuli (Bar et al., 2006; Evans et al., 2012; Huang et al., 2018; Liu & Sumpter, 2018; Mühlenbeck et al., 2016), the presence of GR may have reduced this time because it was quickly recognized and preferred by the observers. So, hypothesizing GR as an affordance, it may be implicitly recognized, facilitating the processing of visual information relating to a stimulus, making aesthetic experience more fluent, in accordance with the “processing fluency theory” for which that fluency could contribute to the pleasantness of the experience associated with the perception of beauty (Witmer, 1893).

It is noteworthy that the GR divides the human stature in a region roughly close to the body center of mass and could even play an important role in human motor behavior (Iosa et al., 2018). It should be noted that the exposition to GR does not regard only the visual system: GR was recently found as a harmonic feature of voluntary and involuntary rhythmic human movements, such as cardiac (Yetkin et al., 2013), respiratory (Iared et al., 2016), and walking rhythms (Iosa et al., 2013; Iosa et al., 2016; Iosa et al., 2019; Serrao et al., 2017). In fact, walking performed with GR between gait phases allows humans to harmonize locomotor acts (Iosa et al., 2016; Iosa et al., 2019), thus reducing energy expenditure (Serrao et al., 2017). Furthermore, it has been demonstrated that perception of internal gait rhythm may be modulated (De Bartolo, Belluscio, et al., 2020a) and that the harmonic structure of music can ameliorate harmony of walking in patients with Parkinson's disease (De Bartolo, Morone, et al., 2020b), especially if acoustic stimulation presents GR harmony (Belluscio et al., 2021), confirming the possibility to consider GR as an affordance to exploit by the observer/listener/acting subject.

However, we must bracket this proposal with some caveats. The main limitation of Experiment 1 was the online administration of the questionnaire that did not allow for controlling some variables such as the eyes' positions with respect to the screen, the response time of the subjects, or their attention level. For example, the questionnaire was formed by 51 items, and it might have led to a progressive physiological decline in the participants' attention due to fatigue.

However, the online administration allowed for enrolling a wide sample of subjects (*N* = 256) that could have attenuated the above‐mentioned limits. Experiment 2 was performed in more controlled conditions, and its results confirmed those obtained in Experiment 1. Subjects were required to judge the most liked stimulus, without any investigations related to the judgment of naturalness, so caution is needed in discussing the role of naturalness of stimuli and its relationship to the preference for stimuli originally or not in GR.

Finally, in our study we did not manipulate canonical orientation of stimuli, so we included only vertical stimuli for anthropomorphic figures and horizontal for images of artworks. Further studies should investigate the possible differences in the GR preferences for vertical versus horizontal stimuli, and these results could contribute to understand the relationship between GR and visual scan process.

## CONCLUSIONS

The results of this study showed a general preference for GR, although not for all types of stimuli; the second one showed that eye fixations were concentrated in the areas related to GR and, when stimuli are in that proportion, a higher aesthetic judgment was correlated with a lower dwell time. The GR has some features analogous to symmetry: both are geometric characteristics widely present in nature (Iosa et al., 2018), including physiological rhythms (Belluscio et al., 2021; Iared et al., 2016; Iosa et al., 2013; Iosa et al., 2016; Iosa et al., 2019; Serrao et al., 2017; Yetkin et al., 2013), both may facilitate the visual processing, hence both could be considered as ecological affordances (Bar et al., 2006; Belluscio et al., 2021; Burrell, 1932; Evans et al., 2012; Gibson, 2014; Huang et al., 2018; Iared et al., 2016; Iosa et al., 2013; Iosa et al., 2016; Iosa et al., 2019; Liu & Sumpter, 2018; Lobo et al., 2018; Serrao et al., 2017; Yetkin et al., 2013), and may be related to the perception of beauty, as artists have well known since ancient time.

## CONFLICT OF INTEREST

The authors declare no potential conflicts of interest with respect to the research, authorship, and/or publication of this article.

## References

[pchj505-bib-0001] Angier, R. P. (1903). *The aesthetics of unequal division* (Doctoral dissertation, Harvard University).

[pchj505-bib-0002] Bar, M. , Neta, M. , & Linz, H. (2006). Very first impressions. Emotion, 6(2), 269–278. 10.1037/1528-3542.6.2.269 16768559

[pchj505-bib-0003] Bastioni, M. , Re, S. , & Misra, S. (2008, January). Ideas and methods for modeling 3D human figures: The principal algorithms used by MakeHuman and their implementation in a new approach to parametric modeling. In Proceedings of the 1st Bangalore annual compute conference (p. 10). ACM. 10.1145/1341771.1341782

[pchj505-bib-0004] Bejan, A. (2009). The golden ratio predicted: Vision, cognition and locomotion as a single design in nature. International Journal of Design and Nature and Ecodynamics, 4(2), 97–104. 10.2495/DNE-V4-N2-97-104

[pchj505-bib-0005] Belluscio, V. , Iosa, M. , Vannozzi, G. , Paravati, S. , & Peppe, A. (2021). Auditory Cue based on the Golden ratio can improve gait patterns in people with Parkinson's disease. Sensors, 21(3), 911. 10.3390/s21030911 PMC7866385 33573043

[pchj505-bib-0006] Benjafield, J. , & Adams‐Webber, J. (1976). The golden section hypothesis. British Journal of Psychology, 67(1), 11–15. 10.1111/j.2044-8295.1976.tb01492.x

[pchj505-bib-0007] Berlyne, D. E. (1970). The golden section and hedonic judgments of rectangles: A cross‐cultural study. Sciences de l'art/Scientific Aesthetics, 7(1), 1–6. 10.2307/1422021

[pchj505-bib-0008] Boselie, F. (1984). The aesthetic attractivity of the golden section. Psychological Research Psychologische Forschung, 45(4), 367–375. 10.1007/BF00309712

[pchj505-bib-0009] Bundgaard, P. F. (2015). Feeling, meaning, and intentionality—A critique of the neuroaesthetics of beauty. Phenomenology and the Cognitive Sciences, 14(4), 781–801. 10.1007/s11097-014-9351-5

[pchj505-bib-0010] Bundgaard, P. F. , Heath, J. , & Østergaard, S. (2017). Aesthetic perception, attention, and non‐genericity: How artists exploit the automatisms of perception to construct meaning in vision. Cognitive Semiotics, 10(2), 91–120. 10.1515/cogsem-2017-0011

[pchj505-bib-0011] Burrell, P. S. (1932). Man the measure of all things: Socrates versus Protagoras (I). Philosophy, 7(25), 27–41. https://www.jstor.org/stable/3747050

[pchj505-bib-0012] Chassy, P. , Lindell, T. A. , Jones, J. A. , & Paramei, G. V. (2015). A relationship between visual complexity and aesthetic appraisal of car front images: An eye‐tracker study. Perception, 44(8–9), 1085–1097. 10.1177/0301006615596882 26562922

[pchj505-bib-0013] Chenier, T. , & Winkielman, P. (2018). The origins of aesthetic pleasure: Processing fluency and affect in judgment, body, and the brain. In Neuroaesthetics (pp. 275–289). Routledge.

[pchj505-bib-0014] Danikas, D. , & Panagopoulos, G. (2004). The golden ratio and proportions of beauty. Plastic and Reconstructive Surgery, 114(4), 1009. 10.1097/01.PRS.0000138702.13724.26 15468417

[pchj505-bib-0015] Davis, F. C. (1933). Aesthetic proportion. The American Journal of Psychology, 45(2), 298–302. 10.2307/1423158

[pchj505-bib-0016] Davis, S. T. , & Jahnke, J. C. (1991). Unity and the golden section: Rules for aesthetic choice? The American Journal of Psychology, 104(2), 257–277. 10.2307/1423158

[pchj505-bib-0017] Davis, T. A. , & Altevogt, R. (1979). Golden mean of the human body. Fibonacci Quarterly, 17(1979), 340–344.

[pchj505-bib-0018] De Bartolo, D. , Belluscio, V. , Vannozzi, G. , Morone, G. , Antonucci, G. , Giordani, G. , Santucci, S. , Resta, F. , Marinozzi, F. , Bini, F. , Paolucci, S. , & Iosa, M. (2020a). Sensorized assessment of dynamic locomotor imagery in people with stroke and healthy subjects. Sensors, 20(16), 4545. 10.3390/s20164545 32823786 PMC7472606

[pchj505-bib-0019] De Bartolo, D. , Morone, G. , Giordani, G. , Antonucci, G. , Russo, V. , Fusco, A. , Marinozzi, F. , Bini, F. , Spitoni, G. F. , Paolucci, S. , & Iosa, M. (2020b). Effect of different music genres on gait patterns in Parkinson's disease. Neurological Sciences, 41(3), 575–582. 10.1007/s10072-019-04127-4 31713758

[pchj505-bib-0020] de Campos, D. , Malysz, T. , Bonatto‐Costa, J. A. , & Oxley da Rocha, A. (2015). More than a neuroanatomical representation in the creation of Adam by Michelangelo Buonarroti, a representation of the Golden ratio. Clinical Anatomy, 28(6), 702–705. 10.1002/ca.22580 26182895

[pchj505-bib-0021] Di Dio, C. , Canessa, N. , Cappa, S. F. , & Rizzolatti, G. (2011). Specificity of esthetic experience for artworks: An fMRI study. Frontiers in Human Neuroscience, 5, 139. 10.3389/fnhum.2011.00139 22121344 PMC3220187

[pchj505-bib-0022] Di Dio, C. , Macaluso, E. , & Rizzolatti, G. (2007). The golden beauty: Brain response to classical and renaissance sculptures. PLoS One, 2(11), e1201. 10.1371/journal.pone.0001201 18030335 PMC2065898

[pchj505-bib-0023] Evans, D. W. , Orr, P. T. , Lazar, S. M. , Breton, D. , Gerard, J. , Ledbetter, D. H. , Janosco, K. , Dotts, J. , & Batchelder, H. (2012). Human preferences for symmetry: Subjective experience, cognitive conflict and cortical brain activity. PLoS One, 7(6), e38966. 10.1371/journal.pone.0038966 22720004 PMC3374766

[pchj505-bib-0024] Eysenck, H. J. , & Tunstall, O. (1968). La personnalité et l'esthétique des formes simples [Personality and aesthetics of simple forms]. Sciences de l' Art/Scientific Aesthetics, 5, 3–9.

[pchj505-bib-0025] Fechner, G. T. (1865). Über die Frage des goldenen Schnitts [about the question of the golden section]. Archiv fur diezeichnenden Kunste, 11, 100–112.

[pchj505-bib-1001] Fechner, G. T. (1890). Wissenschaftliche briefe von Gustav Theodor Fechner u. W. Preyer [Scientific letters from Gustav Theodor Fechner and W. Preyer], 1st edn. Verlag v. L. Voss.

[pchj505-bib-0026] Fink, B. , & Penton‐Voak, I. (2002). Evolutionary psychology of facial attractiveness. Current Directions in Psychological Science, 11(5), 154–158. 10.1111/1467-8721.00190

[pchj505-bib-0027] Fisher, C. B. , & Bornstein, M. H. (1982). Identification of symmetry: Effects of stimulus orientation and head position. Perception & Psychophysics, 32(5), 443–448. 10.3758/BF03202774 7162945

[pchj505-bib-0028] Gamwell, L. (2015). Mathematics and art: A cultural history. Princeton University Press.

[pchj505-bib-0029] Gibson, J. J. (2014). The ecological approach to visual perception: Classic edition. Psychology Press. 10.4324/9781315740218

[pchj505-bib-0030] Godkewitsch, M. (1974). The ‘golden section’: An artifact of stimulus range and measure of preference. The American Journal of Psychology, 43(1), 269–277. 10.2307/1422021 4451211

[pchj505-bib-0031] Green, C. D. (1995). All that glitters: A review of psychological research on the aesthetics of the golden section. Perception, 24(8), 937–968. 10.1068/p240937 8848362

[pchj505-bib-0032] Haines, T. H. , & Davies, A. E. (1904). The psychology of aesthetic reaction to rectangular. Psychological Review, 11, 249–281. 10.1037/h0076096

[pchj505-bib-0033] Hernández‐Castro, F. (2007). Study of principles and methods of proportion (Doctoral dissertation, Universität Duisburg‐Essen, Fakultät für Geisteswissenschaften» Institut für Kunst und Kunstwissenschaft» Institut für Kunst‐und Designwissenschaften [IKUD]).

[pchj505-bib-0034] Huang, Y. , Xue, X. , Spelke, E. , Huang, L. , Zheng, W. , & Peng, K. (2018). The aesthetic preference for symmetry dissociates from early‐emerging attention to symmetry. Scientific Reports, 8(1), 6263. 10.1038/s41598-018-24558-x 29674652 PMC5908848

[pchj505-bib-0035] Iared, V. G. , de Oliveira, H. T. , & Payne, P. G. (2016). The aesthetic experience of nature and hermeneutic phenomenology. The Journal of Environmental Education, 47(3), 191–201. 10.1080/00958964.2015.1063472

[pchj505-bib-0036] Iosa, M. (2018). Il numero meraviglioso: la sezione aurea. Bellezza della matematica, armonia dell'universo, musica della natura [The astonishing number: the golden ratio. Beauty of mathematics, harmony of the universe, music of nature]. Edited by Tangram Scientific Edition, 1st edition.

[pchj505-bib-0037] Iosa, M. , De Bartolo, D. , Morone, G. , Boffi, T. , Mammucari, E. , Vannozzi, G. , Bini, F. , Marinozzi, F. , Antonucci, G. , & Paolucci, S. (2019). Gait phase proportions in different locomotion tasks: The pivot role of golden ratio. Neuroscience Letters, 699, 127–133. 10.1016/j.neulet.2019.01.052 30710663

[pchj505-bib-0038] Iosa, M. , Fusco, A. , Marchetti, F. , Morone, G. , Caltagirone, C. , Paolucci, S. , & Peppe, A. (2013). The golden ratio of gait harmony: Repetitive proportions of repetitive gait phases. BioMed Research International, 2013, 918642. 10.1155/2013/918642 23862161 PMC3687768

[pchj505-bib-0039] Iosa, M. , Morone, G. , Bini, F. , Fusco, A. , Paolucci, S. , & Marinozzi, F. (2016). The connection between anthropometry and gait harmony unveiled through the lens of the golden ratio. Neuroscience Letters, 612, 138–144. 10.1016/j.neulet.2015.12.023 26700875

[pchj505-bib-0040] Iosa, M. , Morone, G. , & Paolucci, S. (2018). Phi in physiology, psychology and biomechanics: The golden ratio between myth and science. Biosystems, 165, 31–39. 10.1016/j.biosystems.2018.01.001 29317314

[pchj505-bib-0041] Kong, F. , Zhang, Y. , Tian, Y. , Fan, C. , & Zhou, Z. (2015). Self‐relevant beauty evaluation: Evidence from an event‐related potentials study. Journal of Integrative Neuroscience, 14(01), 85–95. 25519777 10.1142/S0219635215500028

[pchj505-bib-0042] Kumar, M. , & Garg, N. (2010). Aesthetic principles and cognitive emotion appraisals: How much of the beauty lies in the eye of the beholder? Journal of Consumer Psychology, 20(4), 485–494. 10.1016/j.jcps.2010.06.015

[pchj505-bib-0043] Kwart, D. G. , Foulsham, T. , & Kingstone, A. (2012). Age and beauty are in the eye of the beholder. Perception, 41(8), 925–938. 10.1068/p7136 23362670

[pchj505-bib-0044] Liu, Y. , & Sumpter, D. J. (2018). Is the golden ratio a universal constant for self‐replication? PLoS One, 13(7), e0200601. 10.1371/journal.pone.0200601 30011316 PMC6047800

[pchj505-bib-0045] Lobo, L. , Heras‐Escribano, M. , & Travieso, D. (2018). The history and philosophy of ecological psychology. Frontiers in Psychology, 9, 2228. 10.3389/fpsyg.2018.02228 30555368 PMC6280920

[pchj505-bib-0046] Mach, E. (1883/1912] 1988). In R. Wahsner & H.‐H. von Borzeszkowski (Eds.), Die Mechanik in ihrer Entwicklung historisch‐kritisch dargestellt [The mechanics in their development presented historically and critically] (7th ed.). Akademie‐ Verlag. See McCormack (tr.) [1893/1960] 1974.

[pchj505-bib-0047] Mach, E. (1905/1926] 2002). Erkenntnis und Irrtum: Skizzen zur Psychologie der Forschung, [Knowledge and Error: Sketches for the Psychology of Research]. Reprint of the 5th edn., edited by Martin Eberhardt. Parerga. See McCormack and Foulkes (trs.) 1976.

[pchj505-bib-0049] McManus, I. C. (1980). The aesthetics of simple figures. British Journal of Psychology, 71(4), 505–524. 10.1111/j.2044-8295.1980.tb01763.x 7437674

[pchj505-bib-0050] McManus, I. C. , Cook, R. , & Hunt, A. (2010). Beyond the golden section and normative aesthetics: Why do individuals differ so much in their aesthetic preferences for rectangles? Psychology of Aesthetics, Creativity, and the Arts, 4(2), 113–126. 10.1037/a0017316

[pchj505-bib-0051] Mühlenbeck, C. , Liebal, K. , Pritsch, C. , & Jacobsen, T. (2016). Differences in the visual perception of symmetric patterns in orangutans (Pongo pygmaeus abelii) and two human cultural groups: A comparative eye‐tracking study. Frontiers in Psychology, 7, 408. 10.3389/fpsyg.2016.00408 27065184 PMC4811873

[pchj505-bib-0052] Nienstedt, C. W., Jr. , & Ross, S. (1951). Preferences for rectangular proportions in college students and the aged. The Pedagogical Seminary and Journal of Genetic Psychology, 78(2), 153–158. 10.1080/08856559.1951.10533573 14861401

[pchj505-bib-0053] Piehl, J. (1976). The ‘golden section’: An artifact of stimulus range and demand characteristics. Perceptual and Motor Skills, 43(1), 47–50. 10.2466/pms.1976.43.1.47 958835

[pchj505-bib-0054] Ramachandran, V. S. , & Hirstein, W. (1999). The science of art: A neurological theory of aesthetic experience. Journal of Consciousness Studies, 6(6–7), 15–51.

[pchj505-bib-0055] Reber, R. (2012). Processing fluency, aesthetic pleasure, and culturally shared taste. In A. P. Shimamura & S. E. Palmer (Eds.), Aesthetic science: Connecting minds, brains, and experience (pp. 223–249). Oxford University Press.

[pchj505-bib-0056] Regmi, P. R. , Waithaka, E. , Paudyal, A. , & van Teijlingen, E. (2016). Guide to the design and application of online questionnaire surveys. Nepal Journal of Epidemiology, 6(4), 640–644. 10.3126/nje.v6i4.17258 28804676 PMC5506389

[pchj505-bib-0057] Russell, P. A. (2000). Testing the aesthetic significance of the golden‐section rectangle. Perception, 29(12), 1413–1422. 10.1068/p3037 11257965

[pchj505-bib-0058] Serrao, M. , Chini, G. , Iosa, M. , & Draicchio, F. (2017). Harmony as a convergence attractor that minimizes the energy expenditure and variability in physiological gait and the loss of harmony in cerebellar ataxia. Clinical biomechanics, 48, 15–23. 10.1016/j.clinbiomech.2017.07.001 28704694

[pchj505-bib-0059] Thompson, G. G. (1946). The effect of chronological age on aesthetic preferences for rectangles of different proportions. Journal of Experimental Psychology, 36(1), 50–58. 10.1037/h0054675 21015341

[pchj505-bib-0060] Wever, E. G. , & Zener, K. E. (1928). The method of absolute judgment in psychophysics. Psychological Review, 35(6), 466–493. 10.1037/h0075311

[pchj505-bib-0061] Witmer, L. (1893). *Zur experimentellen Aesthetik einfacher räumlicher Formverhältnisse* [The experimental aesthetics of simple spatial form relationships]. Philosophische Studen, 9, 96144.

[pchj505-bib-0062] Wölfflin, H. (1994). Prolegomena zu einer Psychologie der Architektur, 1886 [Prolegomena to a psychology of architecture, 1886]. In H. F. Mallgrave & E. Ikonomou (Eds.), Empathy, form, and space: Problems in German aesthetics, 1873–1893 (pp. 149–190). Getty Center for the History of Art and the Humanities.

[pchj505-bib-0063] Yetkin, G. , Sivri, N. , Yalta, K. , & Yetkin, E. (2013). Golden ratio is beating in our heart. International Journal of Cardiology, 168(5), 4926–4927. 10.1016/j.ijcard.2013.07.090 23890853

[pchj505-bib-0064] Zeising, A. (1855). Aesthetische Forschungen [Aesthetic research]. Medinger.

[pchj505-bib-0065] Zeki, S. (1999a). Inner vision: An exploration of art and the brain. Oxford University Press.

[pchj505-bib-0066] Zeki, S. (1999b). Art and the brain. Journal of Consciousness Studies, 6, 76–96.

[pchj505-bib-0067] Zhang, Y. , Luo, N. , Hong, F. F. , Yang, C. H. , Xie, Y. F. , Wu, J. Y. , Wang, G. X. , Zhao, P. Q. , Chen, J. W. , & Aashiq, K. (2020). Attractiveness‐related recognition bias captures the memory of the beholder. Journal of Integrative Neuroscience, 19(4), 629–639. 10.31083/j.jin.2020.04.166 33378837

[pchj505-bib-0068] Zhang, Z. , & Deng, Z. (2012). Gender, facial attractiveness, and early and late event‐related potential components. Journal of Integrative Neuroscience, 11(4), 477–487. 10.1142/S0219635212500306 23351053

